# Erosion of the Teeth

**Published:** 1858-04

**Authors:** 


### EDITORIAL DEPARTMENT.

*Erosion of the Teeth.*—In the December number (1857) of the Dental Register of the West, a writer over the initial W. regrets that he cannot agree with the senior editor in his opinion with regard to congenital erosion of the teeth, believing the theory to be "evidently unsound." Now we did not suppose for a moment, that any one of the present day, however limited his observation may have been, doubted so well an established fact. We do not claim the theory as our own; it is older than we are, but we have had frequent opportunities of verifying the truth of it. W. asks, "Did Professor H. ever find this erosion *before* the eruption of the teeth from the gums," adding, "Till this is done his conclusion is based on a mere inference, and one that is doubtfully deducible from the premises." Many of the theories in general pathology are based upon no better foundation than this, and however justifiable we might be in receiving as satisfactory the inference drawn as it is from so strong a presumption, as conclusive of the correctness of the one in question, we are by no means reduced to such necessity. But before we proceed to cite facts, it may be well to refer to a few of the authorities which we have upon the subject, lest they may have escaped the notice of W.

Bunon is of the opinion that congenital erosion of the teeth is occasioned by the action of a humore which insinuates itself into the alveolus (the sac or matrix of the tooth we presume he means) while the recently enameled tooth is still locked up in the alveolar border.* The explanation however, given by this author, of the manner in which the disease is produced is not very clear, but it must be remembered that at the time he wrote, dental pathology and physiology were both in their infancy. Nevertheless, that he was familiar with the occurrence of the disease is very manifest.

Bourdet, a later writer, ascribes it to the action of the liquor contained in the dental sac, which, as a consequence of severe constitutional disease, has become acidulated.† As an acute and accurate observer, as well as a man of science and an experienced practitioner, Bourdet held a deservedly high rank, and would not be likely to err with regard to a matter with which he professes to be well acquainted, and seems to have studied very carefully.

Delabarre says that congenital erosion "is the result of the corroding action of the mucous fluids, in the midst of which the crown of the tooth unfolds itself, and the properties of which vary according to the various states of health and disease."‡ We might extend our quotations from the last named author, but the above is sufficient to show that he regarded the disease in question as an established fact, and, as that is the only point in relation to the matter which our anonymous friend seems to doubt, it is not necessary in this place to enlarge upon the subject.

Maury, Lefoulon, and others, speak of the occurrence of erosion of the teeth during the formation of the enamel, and of course previously to the eruption of the organs.

Thus it is seen that the doctrine of congenital erosion of the teeth,

*Essay Sur Les Maladies Des Dents, page 61.—1743.

† Recherches Et Observations Sur Toutes Les Parties De L'Art Du Dentiste, page 79—Tome 1.—1757.

‡ Traité de la Seconde Dentition, p. 21—1819.

did not originate with the senior editor, but that it has prevailed for more than a hundred years, and has, during this time been verified by the observations of many of our best writers.

So much for authorities. Now, if W. still remains incredulous, and is unwilling to accept the theory on the testimony of others, we can present him with facts that leave no room for doubt upon the subject. We have two jaws, in each of which the crowns of twelve of the permanent teeth in a dentinified and enameled condition, are still inclosed in their alveolar cells, and nearly all of these are more or less marked by erosion. Even the very cusps of several of them are at one or more points, seriously injured by erosion. With such facts before us, the inference to which W. objects, is, we think, fully justifiable. We have also met with many examples of erosion in practice, which to our mind, were as obviously congenital as the above. We have seen some cases where the enamel was almost wholly destroyed, but the more recently formed part is, undoubtedly, the part most liable to suffer, and after having reached the dentine, the destructive agent then attacks that, and sometimes penetrates nearly, and occasionally, quite to the pulp-cavity.

With regard to the allusion we made to the manner of the destruction of the roots of the temporary teeth, having described that operation of the animal economy in another place, we only recur to it now to correct a wrong quotation made by W. In referring to the fleshy body developed at the neck of the dental sac, as the tooth begins to advance towards the summit of the gums, he makes us to say that "experiments had shown that the *extraneous*," instead of *carneous** substance. But this theory of the destruction of the roots of the temporary tooth by an exhaled acidulated fluid is not ours, it is maintained by many of our ablest dental physiologists, and, to our mind, is the most plausible one that has as yet been advanced.

As to the agency which hydrochloric acid, supposed to be set free in the evaporation of the surface of the ocean, might have in the production of caries of the teeth among seamen and those who reside along the sea shore, that idea was thrown out as a mere hypothesis—as a conjecture—not as an established fact, but simply with a view of eliciting inquiry upon the subject. In this, so far at least, as words are concerned, we have succeeded. We would be glad, although we do not object to words, to have something better. Experiments alone can

*** See Report of Proceedings in Oct. No. (1857) of Am. Jour. Dent. Sci. p. 482.**

settle the question. We hope some competent person will undertake them.

Time will not permit us at present, to extend our remarks on the views of W. to greater length, as the Register, in which it is published, did not reach us until our printer was making up the last form of the present number of the Journal. It is seldom that we notice the strictures of anonymous writers, but in this instance, we have thought proper to depart from our general rule upon the subject.

We intended to have published the above in our January number, but it was excluded for want of room.

